# Novel Regulators of Sugar-Mediated Lateral Root Development in *Arabidopsis thaliana*

**DOI:** 10.3390/genes11111257

**Published:** 2020-10-25

**Authors:** Jinzhu Li, Bingxin Wang, Xinxing Zhu, Rong Li, Jing Fu, Hongchang Cui

**Affiliations:** 1 State Key Laboratory of Crop Stress Biology for Arid Areas and College of Life Sciences, Northwest A&F University, Yangling, Xianyang 712100, Shaanxi, China; lijinzhu14@mails.ucas.ac.cn (J.L.); wbx2464@163.com (B.W.); zhuxinxing@nwafu.edu.cn (X.Z.); lirong157351@nwafu.edu.cn (R.L.); jingfu@nwsuaf.edu.cn (J.F.); 2 Department of Biological Science, Florida State University, Tallahassee, FL 32306, USA

**Keywords:** lateral root, sugar, EMS mutagenesis, mapping, WOX7, HXK1

## Abstract

Lateral root development is a complex process regulated by numerous factors. An important role for sugar in lateral root development has been known for a while, but the underlying molecular basis still remains unclear. In this study, we first showed that WOX7, a sugar-inducible negative regulator of lateral root development, acts downstream of the glucose sensor HXK1. Using a transgenic line homozygous for a transgene expressing GFP under the control of the *WOX7* promoter, we next performed a genetic screen to identify additional genes in this development pathway. A number of mutants with altered level of *WOX7* expression were recovered, and two with increased *WOX7* expression, named *ewe-1* and *ewe-2* (for *Enhanced WOX7 Expression*), were further characterized. Both mutants manifest delayed lateral root development, and genetic analysis indicates that single recessive mutations are responsible for the observed phenotypes. The mutations were then located to similar regions on chromosome 2 by marker-assisted analyses, and candidate genes were identified through whole genome sequencing. The significance and limitations of this work are discussed.

## 1. Introduction

Root is essential to plant growth and development, because it is responsible for water and nutrient uptake from the soil. As the plant grows, the root expands its surface by generating lateral roots as well as by extending its length. Lateral roots are derived from a subset of cells in the stele through a complex developmental process that is controlled by both internal and environmental factors, including almost all hormones [1,2,3,4], nutrient and water availability [5,6,7,8]. Sugar also plays a role in lateral root development and root growth, as both an energy source and signaling molecule [9]. Synthesized in the shoot, sugar is transported to the root as well as other non-photosynthetic organs, mainly in the form of sucrose, which is converted to glucose that can be directly utilized in energy metabolism [10]. In laboratory assays, sucrose or glucose is generally supplied in the growth medium to enhance plant growth [11]. At lower concentrations (below 1%), sugar promotes root growth and lateral root formation, but becomes inhibitory at higher concentrations [12,13,14].

There exist two signaling pathways for glucose sensing—an HXK1 (hexokinase 1)-dependent pathway and an HXK1-independent pathway [15,16]. In Arabidopsis, there are five HXK1 homologs, including two with catalytic activity, HXK2 and HXK3, and three enzymatically inactive, HKL1, HKL2 and HKL3 [17]. A positive role for HXK1 in lateral root development has been reported previously, as in *hxk1/gin2* mutant, lateral root number is reduced [13]. HKL1 appears to plays a negative role in glucose signaling probably by interfering with HXK1 [18]. To date, the HXK1-independent pathway is still poorly understood [19]. RGS1, a transmembrane protein involved in G-protein signaling, has been shown to be the glucose sensor in the HXK1-independent pathway [20,21]. Although RGS1 seems to interact with the HXK1-dependent pathway in root growth and lateral root development, mutations in RGS1 and related components in the G-protein signaling pathway, such as GPA1 and THF1, do not appear to affect lateral root formation [13]. 

Despite the well-documented role of sugar in lateral root development, very few genes are presently known that modulate the effects of sugar on lateral root development. In a genetic screen for mutants with altered lateral root numbers in high sugar medium, Deak and Malamy identified the *lrd2* mutant that has an elevated density of lateral roots [22]. The gene with the causal mutation was subsequently cloned and found to be a long-chain fatty acid synthase (LACS2), an enzyme involved in cutin biosynthesis [23]. LRD2 does not respond to sugar, but rather slows down sugar uptake by forming a cuticle layer on leaves, thereby mitigating osmotic stress caused by high sugar [23]. Another mutant, *lrd3*, was also identified in the same genetic screen [24]. LRD3 was shown to be a LIM-domain protein of unknown function expressed specifically in phloem companion cells, and its mutation causes morphological abnormality in the phloem [24]. The *lrd3* mutant phenotype is hence attributed to lack of nutrients, rather than a defect in sugar signaling [24].

In a previous study we showed that WOX7, a WUSHEL-related transcription factor, negatively regulates lateral root development in a sugar-dependent manner [25]. In *wox7* mutant, lateral root number increased primarily due to a higher number of lateral root primordia, whereas in *WOX7* overexpressing lines, lateral root number decreased [25]. *WOX7* is expressed throughout all developmental stages of lateral root development, but its expression level increases when sugar concentration increases in growth medium [25]. As the first known example of transcription factors linking sugar response and lateral root development, WOX7 provides an excellent model for dissecting the gene regulatory pathway from sugar sensing to cell division. 

In this report, we first showed that *WOX7* acts downstream of both the HXK1-dependent and independent signaling pathways. We then described a genetic screen for mutants that affect *WOX7* expression in lateral root primordium. Two mutants with elevated levels of *WOX7* expression were identified and further characterized. By genetic segregation analyses and allelism tests, we determined that the lateral root mutant phenotypes in the two mutants are due to different single recessive mutations. Both mutants manifest an increase in lateral root number and density. The causal mutations were first located to a small region on chromosome 2 by marker-assisted crude mapping and then narrowed down to a couple of candidate genes by next-generation whole genome sequencing. 

## 2. Materials and Methods

### 2.1. Plant Growth Conditions

For most experiments, seedlings grown aseptically were used in this study. Seeds were cleaned with 10% bleach, thoroughly washed with sterile H_2_O, and then were allowed to germinate in 1x MS medium containing 1% sucrose in square petri dishes placed vertically in a Percival growth chamber (model 41L). The growth condition is 16-h light (50 micromoles/m2/sec of light irradiance) and 8-h darkness and a constant temperature of 22 °C. Bulking was done in a growth room with the same setting. The transgenic line carrying the GFP reporter gene under the control of the *WOX7* promoter was described previously [25]. 

### 2.2. β-glucuronidase (GUS) Staining and Microscopy

GUS staining was performed essentially as described in the Arabidopsis book [26]. Seedlings were incubated in GUS staining solution overnight at 37 °C. For light microscopy, the roots were first cleared in a drop of chloral hydrate solution (7.5 g chloral hydrate dissolved in 3 mL 50% glycerol) on a glass slide for 1–5 min. Images were captured using an Olympus BX61 compound microscope.

### 2.3. EMS Mutagenesis and Mutant Screening

Mutagenesis was conducted according to the Arabidopsis handbook [26]. Briefly, approximately 20,000 seeds homozygous for the *WOX7pro::GFP* reporter gene in the Columbia background were added to 10 mL 0.1% ethyl methanesulfonate (EMS, Sigma M0880, MO, USA) in a 15 mL conical tube, and the tube was rotated on a rotator overnight at room temperature. After thorough washing with sterile H_2_O, the seeds were suspended in 0.01% agarose and sowed in soil (three to five seeds per pot). At maturity, the seeds from all plants in a pot were pooled.

For mutant screening, 100 seeds from each tube were germinated in Murashige and Skoog medium (MS) medium. Seven days after germination, seedlings were transferred to a MS plate supplemented with 5% glucose and allowed to grow for one more day. For microscopic examination, roots were cut and loaded in H_2_O on a slide, and GFP fluorescence was checked on an Olympus BX61 compound microscope. As a control, seedlings with the same promoter-GFP construct without EMS treatment were grown in a similar manner.

### 2.4. Genetic Analyses

For allelism test, the mutants were crossed to each other, and GFP fluorescence in F1 seeds was compared to that in the wildtype parent line, using an Olympus BX61 compound microscope. To determine the genetic basis of the mutations, the mutants were crossed to wild type plants (Columbia). In the F2 segregating population, the number of mutants and normal plants with GFP fluorescence were counted, and the number of genes involved as well as dominance or recessiveness of the mutations were determined by χ² test.

### 2.5. DNA Extraction, Genotyping and Mapping

To examine *WOX7* expression in the *hxk1* mutant, the transgenic line *WOX7pro::GFP* was crossed to the *gin2-1/hxk1* mutant [15]. DNA was extracted from leaves of 1-month-old plants using the CTAB method, and the *HXK1* genotype was determined by PCR using primers: *gin2-1*_F AGATACTACTAAAGACGAGGAGCTG and *gin2-1*_R AGGTAGAGAGAGTGAGAAGCAGCA.

To generate the F2 segregating population for mapping, the mutants were crossed to L*er*. Mutants and normal plants were then selected based on GFP fluorescence (plants with no GFP fluorescence were excluded), and DNA was extracted from individual plants using the CTAB method. For crude mapping, SSLP markers were amplified by PCR, and the PCR products were resolved in 4% agarose gel. 

For mapping with whole genome sequencing, four types of plants were compared: the parent line homologous for the *WOX7pro::GFP* reporter gene, the mutants from the genetic screen, normal and mutant plants selected from the F2 segregating population, and genome sequencing was done using the Illumina paired-end next generation sequencing method. To increase the resolution, we chose to sequence the mutants and normal plants at 50X coverage, whereas the parent line and the mutants were sequenced at 20X. After cleaning, the reads were aligned to the TAIR10 Arabidopsis genome with Burrows–Wheeler Aligner (S2) [27]. After genome assembly using a commercial service (Wuhan SeqHealth Technology Company, China), SNPs in the mutants were first identified. Variants calling was performed with GATK [28], SNP or InDel was annotated with ANNOVAR [29]. Finally, after SNP/InDel index and delta SNP/InDel index were calculated, SNPs with a SNP-index >0.5 in Ler pool and those with a SNP-index >0.5 in F2 normal pool were removed, and only those with a SNP-index >1 in F2 mutant pool were retained. The raw next generation sequencing data have been deposited at NCBI and can be accessed publicly from https://www.ncbi.nlm.nih.gov/bioproject/PRJNA669402 [30].

## 3. Results

### 3.1. WOX7 Acts Downstream of HXK1 and RGS1

**Figure 1:**
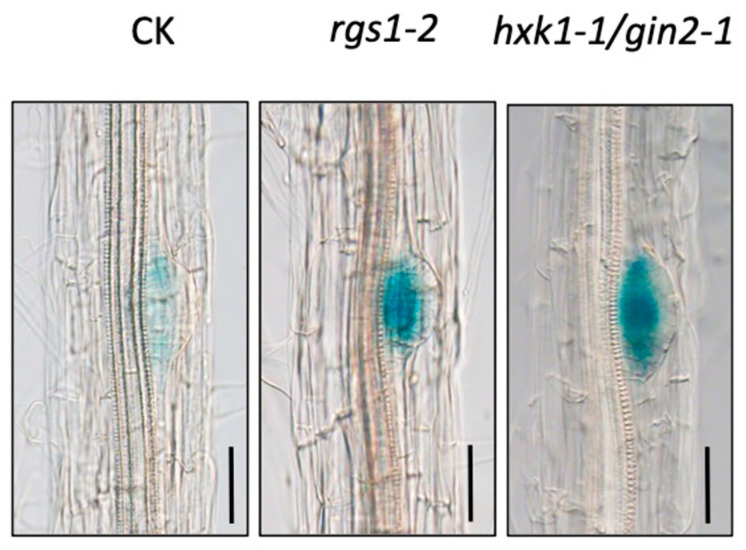
*WOX7* expression increased in the *hxk1* and *rgs1* mutants, as indicated by the blue stain from the *WOX7pro::GUS* reporter gene. CK. *WOX7pro::GUS* in wild type Columbia background. Bars = 50 μm. CK, wild type with the *WOX7pro::GUS* reporter gene. CK and mutants are all in Columbia background. The MS medium contains 1% sucrose, no glucose.

To determine whether *WOX7* is involved in the HXK1-dependent or independent pathway, its expression level and pattern in the *gin2-1/hxk1* and *rgs1-2* mutant were examined using the *WOX7pro::GUS* reporter that we generated previously [25], which was introduced into the two mutants by genetic crossing. As shown in Figure 1, GUS staining was enhanced in both mutants, suggesting that *WOX7* acts downstream of *HXK1* and *RGS1*. This result is intriguing, as HXK1, but not RGS1, was shown to affect lateral root development [13]. Nevertheless, this result indicates that WOX7 is involved in the HXK1-dependent sugar signaling pathway.

### 3.2. A Genetic Screen for Mutants with Altered WOX7 Expression

To identify factors that are involved in the WOX7 developmental pathway, we next performed a genetic screen for mutants that display an altered expression pattern or level of *WOX7*. Approximately 20,000 seeds homogeneous for a transgene that expresses an endoreticulum localized GFP under the control of the *WOX7* promoter [25] were treated with ethyl methanesulfonate (EMS). To increase *WOX7* expression level, thus facilitating GFP fluorescence observation, 5-day-old M2 seedlings germinated in normal MS medium were transferred to MS medium supplemented with 5% glucose and maintained in high sugar medium for 1 day before microscopic examination. Totally, approximately 10,000 M2 seedlings from 1820 pools of seeds were screened.

**Figure 2:**
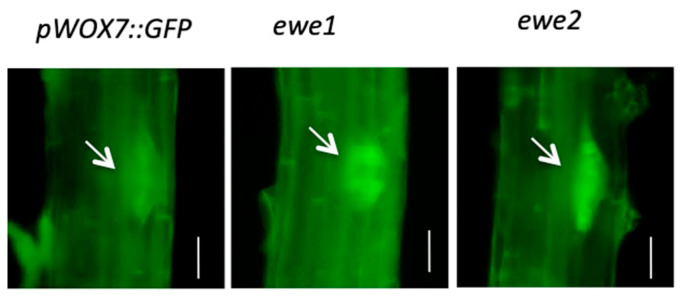
Two mutants with enhanced *WOX7* expression in lateral root primordia (arrows) in 10-day-old seedlings, as shown by green fluorescence from the *WOX7pro::GFP* reporter gene. Bars = 50 μm.

Two types of mutants were anticipated from the screen: those with decreased or elevated GFP fluorescence, because *WOX7* can be regulated positively and negatively. Many putative mutants with increased GFP fluorescence were obtained, but much fewer mutants with lower GFP signals were identified (150 vs. 6). We selected two mutants with an elevated level of *WOX7*, named *Enhanced WOX7 Expression* (*ewe*), in further characterization (Figure 2).

### 3.3. Characteristics of the ewe1 and ewe2 Mutants

**Figure 3:**
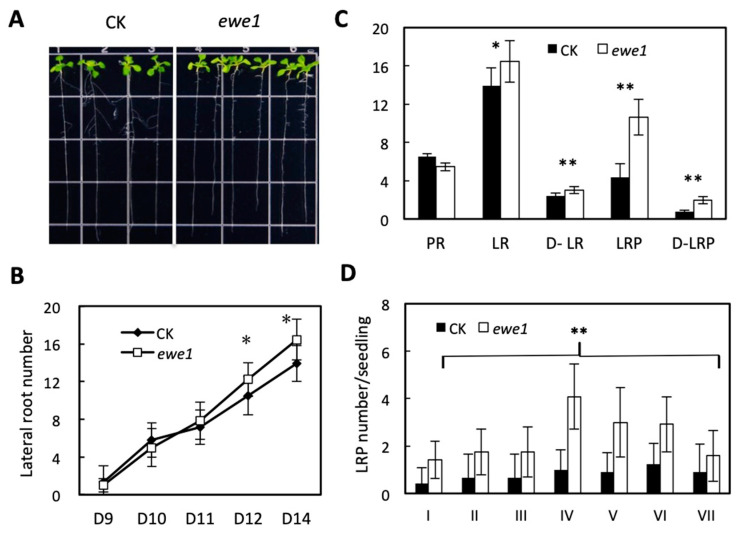
Characteristics of the *ewe1* mutant. (**A**) The *ewe1* mutant and its parental line, 12 days after germination. CK, the parent line containing the *pWOX7pro::GFP* reporter gene in wild type background (Columbia). (**B**) Lateral root number per seedling 9–14 days after germination. *n* = 14. (**C**) Primary root length (PR), lateral root number (LR), lateral root density (D-LR), number of lateral root primordia (LRP), and lateral root primordia density (D-LRP) 14 days after germination. *n* = 14. (**D**) Density of lateral root primordia at various developmental stages in 12-day-old seedlings. *n* = 14. The asterisks indicate degree of significance in the comparison, *t*-test, two-tailed. * *p* < 0.05, ** *p* < 0.01.

**Figure 4:**
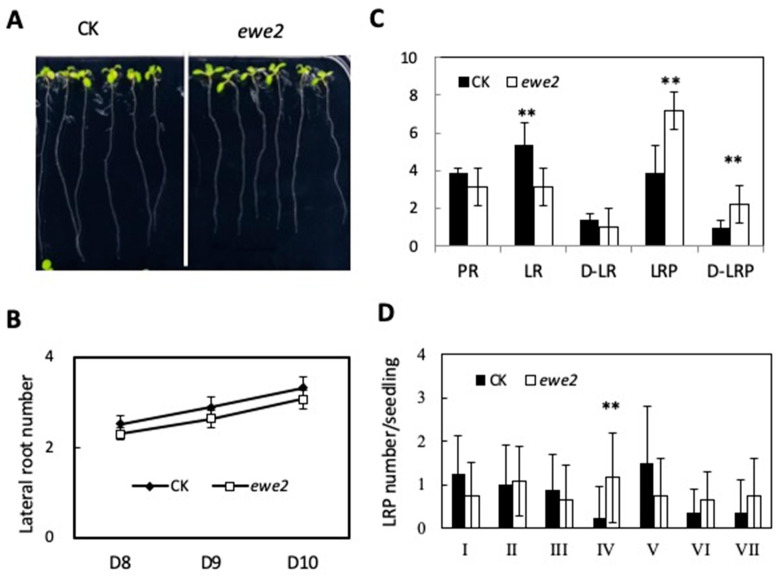
Characterization of the *ewe2* mutant. (**A**) *ewe2* mutant and wild type seedlings, 12 days after germination. CK, the parent line containing the *pWOX7pro::GFP* reporter gene in wild type background (Columbia). (**B**) Lateral root (LR) per seedling from day 9–12 after germination. *n* = 13. (**C**) Primary root length (PR), lateral root numbers (LR), lateral root density (D-LR), lateral root primordia number (LRP), and lateral root primordia density (D-LRP) 14 days after germination. *n* = 13. (**D**) Number of lateral root primordial per seedling at various developmental stages in 12 day-old seedlings. *n* = 12. The asterisks indicate degree of significance in the comparison, *t*-test, two-tailed. * *p* < 0.05, ** *p* < 0.01.

To determine the relationship between the two mutants, we performed allelism test. As seen in Table 1, the results indicate that the causal mutations occur in different genes. Genetic analyses further showed that the two mutated genes are recessive single loci (Table 1). We next examined the effects of these mutants on lateral root development. Compared to the wild type, *ewe1* had a greater number of visible lateral roots, particularly when the seedlings become older (Figure 3A,B), whereas *ewe2* had fewer (Figure 4A,B). Because the primary root length was slightly affected in both mutants (Figure 3A,B and Figure 4A,B), we also calculated lateral root density, which is defined as the number of lateral roots per unit length of primary root. The same trend was observed (Figure 3C and Figure 4C). Interestingly, both mutants showed significantly more lateral root primordia, in terms of either the total number per seedling or per unit of primary root length (Figure 3C and Figure 4C). 

Lateral root development undergoes several morphologically discernable stages [6]. Stage I is the initiation stage whose hallmark is a longitudinal asymmetric cell division in the founder cells; Stage II has two cell layers resulting from periclinal divisions from the initial cell layer; Stage III and IV have three or four cell layers, respectively; Stage V is a multi-cell layer stage; Stage VI is pre-emergence; and VII is post emergence. To determine at which stage lateral root development was affected in the two mutants, we counted the number of lateral root primordia at various stages in the mutant and wild type seedlings. Compared to the wild type, the e*we1* mutant had more lateral root primordia at all stages (Figure 3D), but the *ewe2* mutant appears to affect stage IV only (Figure 4D). These results together suggest that lateral root development was affected in both mutants, during both lateral root primordial development and post-emergence growth, albeit to different degrees.

### 3.4. Mapping the Causal Mutations in the ewe1 and ewe2 Mutants

For initial mapping, we used the marker-assisted mapping method, where Simple Sequence Length Polymorphism (SSLP) was amplified by PCR. The F2 segregation population was generated by crossing the mutants to wild type in the L*er* background, followed by selfing, and mutants were selected based on enhanced GFP fluorescence from the *WOX7pro::GFP* reporter gene. Using 14 SSLP markers evenly distributed on all five chromosomes and 21 mutant plants, we mapped the *ewe1* mutation to a region near the CIW2 marker on chromosome 2 (Table 2, Figure S1). With more markers around CIW2 and 36 additional mutant plants, we further narrowed the mutant locus to an approximately 3 Mbp region spanning the SSLP markers F13J11 to F7E22 (Table 3, Figure S2). For the *ewe2* mutant, 16 SSLP markers and 31 mutants were used in the initial mapping, which mapped the mutation to a region around SSLP markers F504A and CIW2 (Table 4, Figure S3). Further analysis using SSLP markers near F504A and CIW2 and a larger number of mutants (48) showed that the *ewe2* mutation was within a 2Mbp region spanning SSLP markers T4E5 and F10C8 (Table 5, Figure S4). 

To identify the genes with the causal mutations, we adopted a mapping by whole-genome sequencing method [31,32], using the Illumina paired-end sequencing protocol. The mutant plants as described above for the marker-assisted analysis were used for this purpose, as well as three additional groups of plants: normal plants from the F2 segregating populations, the original mutants, and the parent line homologous for the *WOX7pro::GFP* marker line, plus the Ler and Col reference genome. For all samples, more than 92% of reads are of high quality and can be aligned to the Arabidopsis genome (Table S1). 

To determine whether a particular SNP is associated with the mutant phenotype, we first used the Manhattan plot that compares the SNP index in F2 normal plants and F2 mutants. As shown in Figure S5, in both mutants a prominent peak was easily visible on chromosome 2 and in a region corresponding to that as determined by the marker-assisted crude mapping. Because the mutations in both mutants fall in a similar region, this raises the possibility that the *WOX7pro::GFP* marker gene may have been mutated. To rule out this possibility, we searched the mutant genome sequence for the presence of this chimeric construct, which located the reporter gene at 3963972 bp on chromosome 3. Next, through comparative analyses (details described in the methods), we narrowed down the causal mutations to a region that contains a small number of gene and transposable elements (Table S2). In both the *ewe1* and *ewe2* mutants, however, only two genes were found to contain G to A transition mutation in their coding region, which is characteristic of the EMS mutagen. In the *ewe1* mutant, this point mutation is located in AT2G02930 and AT2G18750, and causes change in amino acids (P113L and E522K respectively). In the *ewe2* mutant, the mutated genes are AT2G04040 and AT2G14260, which both have a Valine substituted by Alanine in both proteins (V147A and V215A, respectively). 

To determine which candidate gene is responsible for the lateral root phenotype in each mutant, we next checked the expression of the four candidate genes in Arabidopsis root using the BAR tool (bar.utoronto.ca) [33]. As shown in Figure S6, all candidate genes are expressed in the differentiation zone, where lateral roots form, albeit at different levels and in different cell types. Strikingly, AT2G18750 and AT2G14260 show a preferential expression pattern in lateral root primordium and pericycle, from which lateral roots are derived in *Arabidopsis thaliana* [6]. In contrast, AT2G02930 and AT2G04040 are both expressed in the phloem. It is thus reasoned that AT2G18750 and AT2G14260 are more likely to be the genes that carry the causal mutations in the *ewe1* and *ewe2* mutants respectively.

## 4. Discussion

Sugar is essential to lateral root growth and development, as well as other aspects of plant growth and development, both as an energy source and as a signaling molecule. Although the glucose sensor HXK1 has been shown to be an important regulator of lateral root development, how lateral root development is regulated by sugar is still unclear. In the present study, we first showed that WOX7, a sugar-inducible transcription factor with a negative role in lateral root development [25], acts downstream of HXK1, thus providing a critical piece to this sugar-mediated lateral root development pathway.

Using a transgenic line harboring the GFP reporter gene under the control of the *WOX7* promoter, we next performed a genetic screen seeking other components in the above-mentioned sugar-mediated lateral root development pathway. Two major groups of mutants were expected: one in activators of *WOX7*, which is expected to have reduced GFP fluorescence, and the other in *WOX7* repressors, which would have a higher level of GFP fluorescence. Both groups of mutants were obtained, but most mutants had an elevated level of *WOX7* expression. Two mutants with increased *WOX7* expression, namely *ewe1* and *ewe2*, were selected for further characterization in this study due to their robust changes in GFP fluorescence. Surprisingly, we found that the two mutants displayed opposite phenotypes in terms of lateral root number and density, although both had an elevated level of *WOX7*. One explanation for this difference is that, in addition to affecting *WOX7* expression, the two genes may have other roles in lateral root development. Identification of the genes with the causal mutations will allow us to address this question. Alternatively, the genetic analyses indicate that the mutant phenotypes in *ewe1* and *ewe2* are caused by single recessive mutations, which are located at similar regions on chromosome 2 by marker-assisted mapping. Whole genome sequencing narrowed down the causal mutations to two genes in each mutant, *AT2G02930* and *AT2G18750* in *ewe1*, and *AT2G04040* and *AT2G14260* in *ewe2*. Further analyses of their spatial expression in the root allow us to pin down the candidate genes to *AT2G18750* and *AT2G14260*, although we could not rule out the possibility that the other genes are the actual lateral root determinants. Among these genes, *AT2G18750* has the highest probability of being a regulator of sugar signaling and regulator of *WOX7*, because it encodes a putative calmodulin-binding protein involved in calcium signaling. In contrast, none of the other candidate genes have an apparent role in signaling or transcriptional regulation. *AT2G04040* is a detoxifying efflux carrier for plant-derived antibiotics and other toxic compounds [34], whereas *AT2G14260* is a proline iminopeptidase involved in proteolysis and *AT2G02930* encodes a glutathione S-transferase (*GST16*). Whether and how these genes regulate *WOX7* expression awaits further experimental testing. 

## 5. Conclusions

In this study we found that *WOX7*, a sugar responsive gene, is involved in the HXK1 sugar signaling pathway. Via EMS mutagenesis, we identified two mutants that have enhanced expression of *WOX7* and altered number of lateral roots, which were further characterized. For each mutant, we finally narrowed down the causal mutations to two candidate genes through marker assisted and whole genome sequencing mapping methods. These mutants are instrumental in deciphering a yet uncharacterized developmental pathway that links sugar sensing to lateral root development.

## Figures and Tables

**Table 1:** Genetic analyses of two mutants obtained in the genetic screen.

Cross	Generation	Total Seedlings Analyzed	Mutant Phenotype	Wild Type	ᵪ^2^ (3:1)
*ewe 1* × Col	F2	78	18	60	0.155 ^a^
*ewe2* × Col	F2	64	16	48	0.004 ^a^
*ewe1* × *ewe2*	F1	10	—	10	

^a^ Not significant at *p* = 0.05.

**Table 2:** Marker-assisted mapping of *ewe1* (1).

Chromosome	SSLP Marker	Distance to Mutation (cM)
1	YUP8H12	64.3
1	T17H3	54.8
1	NGA128	42.9
2	CIW2	19
2	NGA168	31
3	CIW11	52.4
3	T32N15A	50
3	NGA6	38.1
4	CIW5	42.9
4	FCA8	33.3
4	F17I5A	38.1
4	NGA1107	40.5
5	NGA151	40.5
5	CIW9	40.5

cM, centiMorgan.

**Table 3:** Marker-assisted mapping of *ewe1* (2).

Chromosome	SSLP Marker	Distance to Mutation (cM)
2	T18E12	16.67
2	F15L11	11.11
2	F7E22	6.94
2	T4E5	6.94
2	F13J11	6.94
2	F26C24	9.72
2	NGA168	34.72

cM, centiMorgan.

**Table 4:** Marker-assisted mapping of *ewe2* (1).

Chromosome	SSLP Marker	Distance to Mutation (cM)
1	YUP8H12	40
1	T17H3	54.8
1	NGA128	54.8
2	F504A	25.8
2	CIW2	25.8
2	CIW3	29
2	NGA1126	45.1
2	NGA168	40.3
3	F14P3	45.1
3	NGA162	54.8
3	CIW11	48.3
3	NGA6	58
4	CIW5	40.9
4	T32N4A	50
4	CIW6	54.8
4	FCA8A	59.6
5	NGA151	50
5	CIW9	53.2

cM, centiMorgan.

**Table 5:** Marker-assisted mapping of *ewe2* (2).

Chromosome	SSLP Marker	Distance to Mutation (cM)
2	F15L11	5.21
2	T12H3	4.17
2	F7E22	4.17
2	T4E5	3.13
2	F10C8	3.13
2	T17A11	4.17

cM, centiMorgan.

## Supplementary Materials

The following are available online at https://www.mdpi.com/2073-4425/11/11/1257/s1, Figure S1: Initial mapping of the causal mutation in *ewe1.* Figure S2: Refined mapping of the causal mutation in *ewe1.* Figure S3: Initial mapping of the causal mutation in *ewe2.* Figure S4: Refined mapping of the causal mutation in *ewe2*. Figure S5: Manhattan plots showing the approximate locations (arrows) of the causal SNPs in *ewe1* and *ewe2* mutants. Figure S6: Digital spatial expression pattern of candidate genes in root. Table S1: Summary of whole genome sequencing for mapping. Table S2: Candidate genes and mutation types in the *ewe1* and *ewe2* mutants.
