# Supplementary material for: Novel Regulators of Sugar-Mediated Lateral Root Development in Arabidopsis thaliana
Source: Genes (Basel). 2020 Oct 25;11(11):1257. doi: 10.3390/genes11111257 (PMC7716218; doi:10.3390/genes11111257)
Supplement: Supplementary file 1 [file genes-11-01257-s001.zip › Li et al suppl figures.pdf]

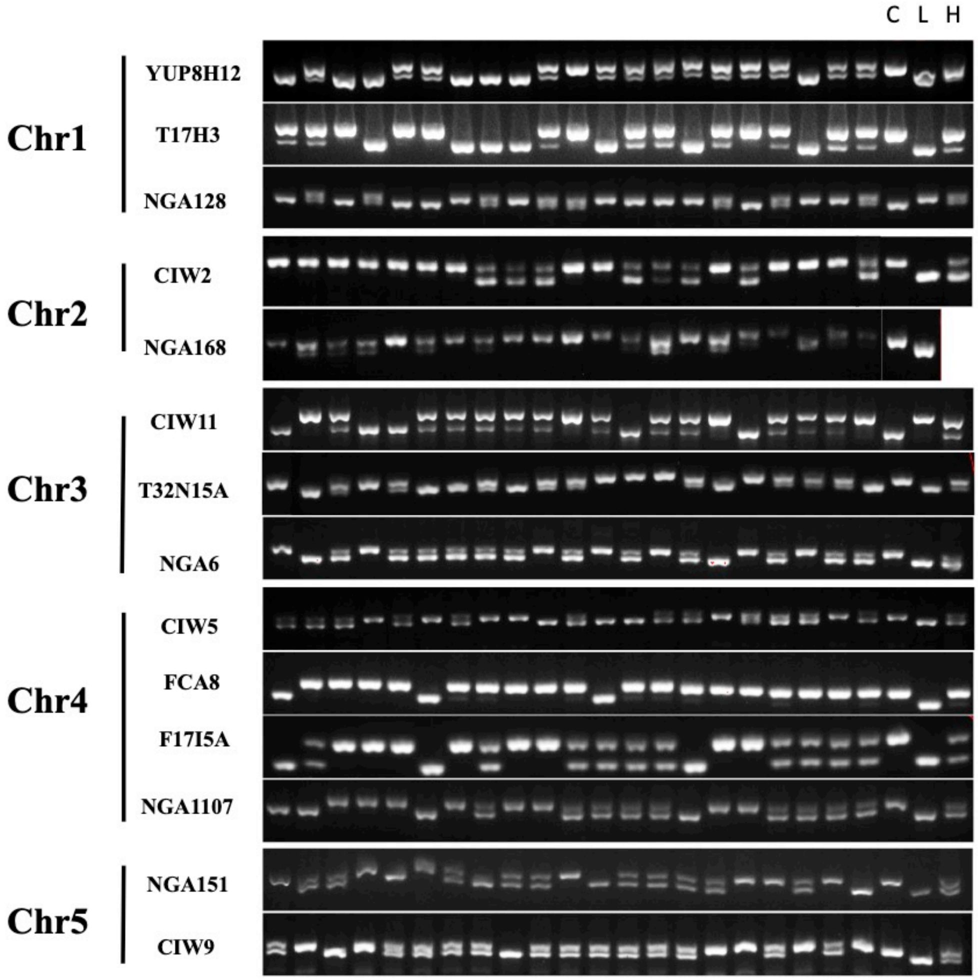

Fig S1. Initial mapping of the causal mutation in *ewe1*.
C. Columbia; L, L*er*; H, mix of Columbia and L*er* DNA.

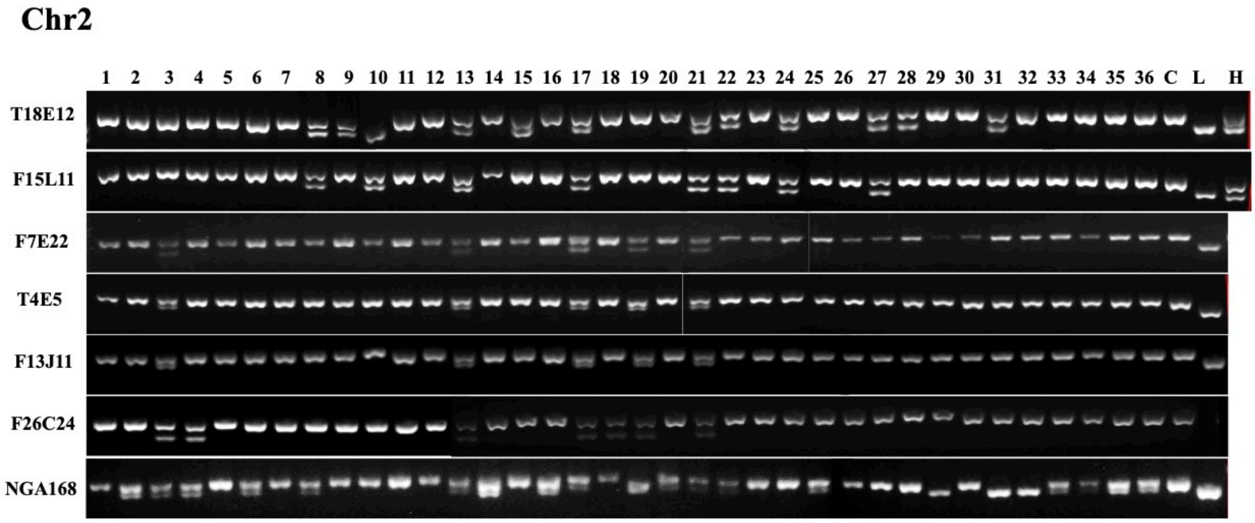

Fig S2. Refined mapping of the causal mutation in *ewe1*.
C. Columbia; L, L*er*; H, mix of Columbia and L*er* DNA.

F15L11

T16I21

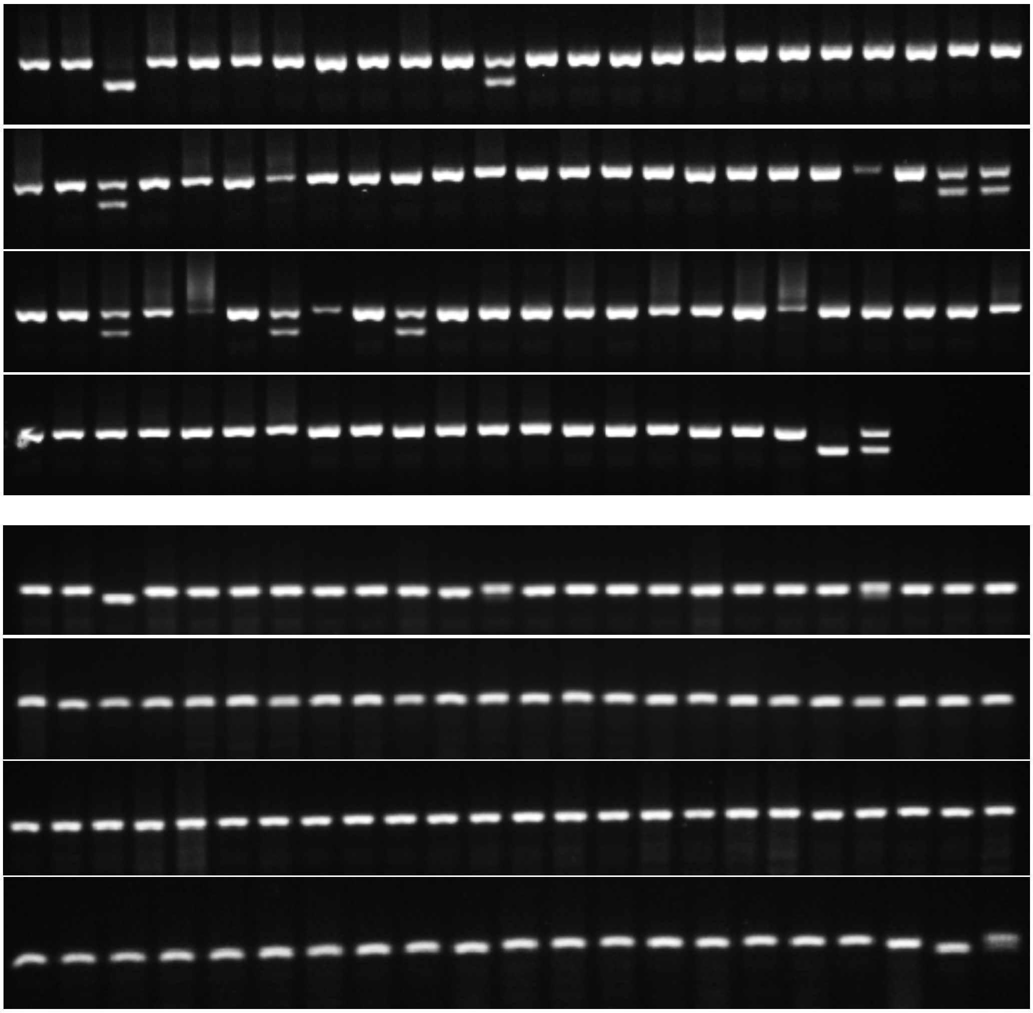

Fig S3. Initial mapping of the causal mutation in *ewe2.*
C. Columbia; L, L*er*; H, mix of Columbia and L*er* DNA.

F7E22

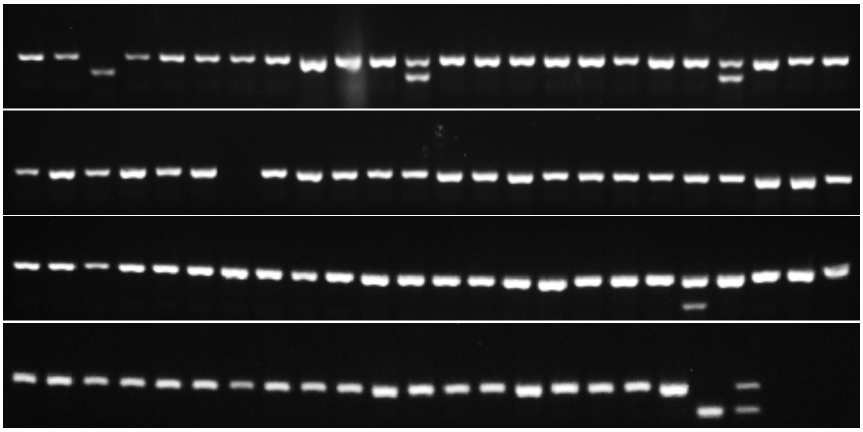

F26C24

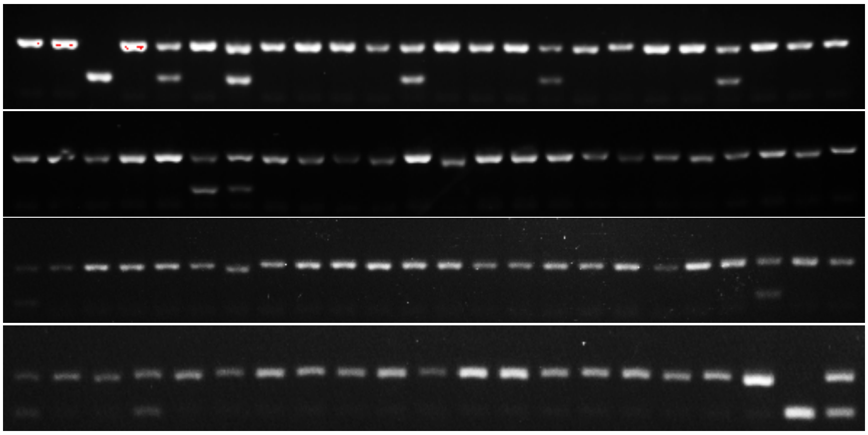

Fig S4. Refined mapping of the causal mutation in *ewe2*.
C. Columbia; L, L*er*; H, mix of Columbia and L*er* DNA.

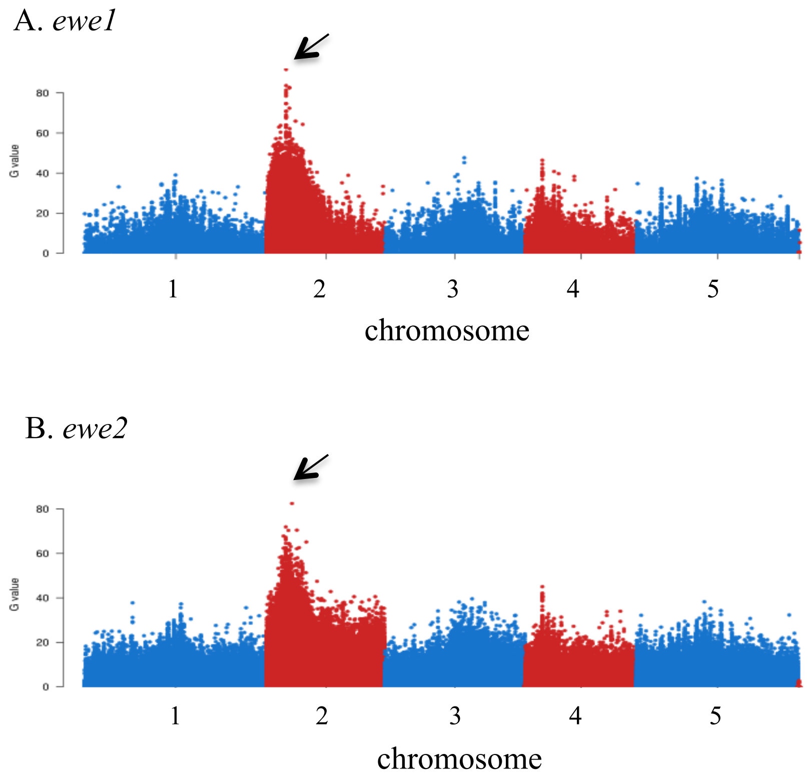

Fig S5. Manhattan plots showing the approximate locations (arrows) of the causal SNPs in *ewe1* and *ewe2* mutants. The comparison was between F2 normal plants and F2 mutants.

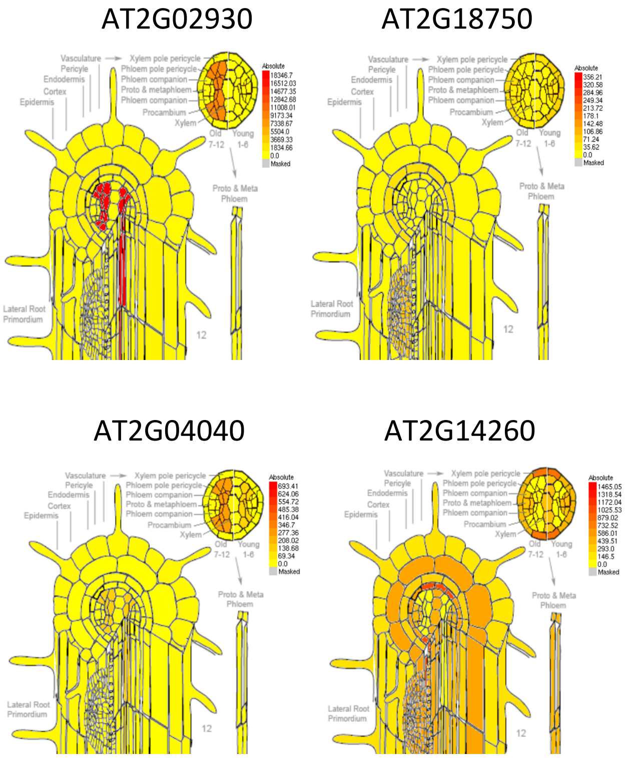

Fig S6. Digital spatial expression pattern of candidate genes in root, according to the eFP tool (Bar.utoronto.ca). Shown in the right of each image is the color code for gene expression level.
